# Supplementary material for: Analysis of gene expression within individual cells reveals spatiotemporal patterns underlying Vibrio cholerae biofilm development
Source: PLoS Biol. 2025 May 16;23(5):e3003187. doi: 10.1371/journal.pbio.3003187 (PMC12121927; doi:10.1371/journal.pbio.3003187)

S2A, non-corrected data

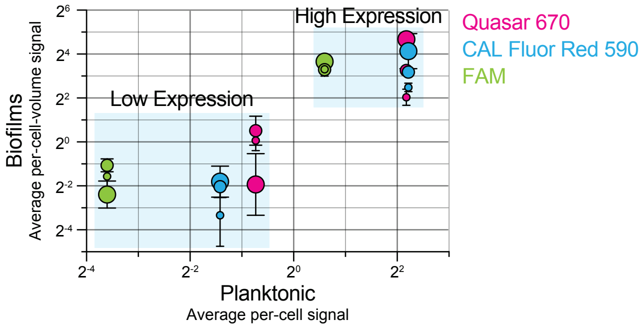

S3B, non-corrected data

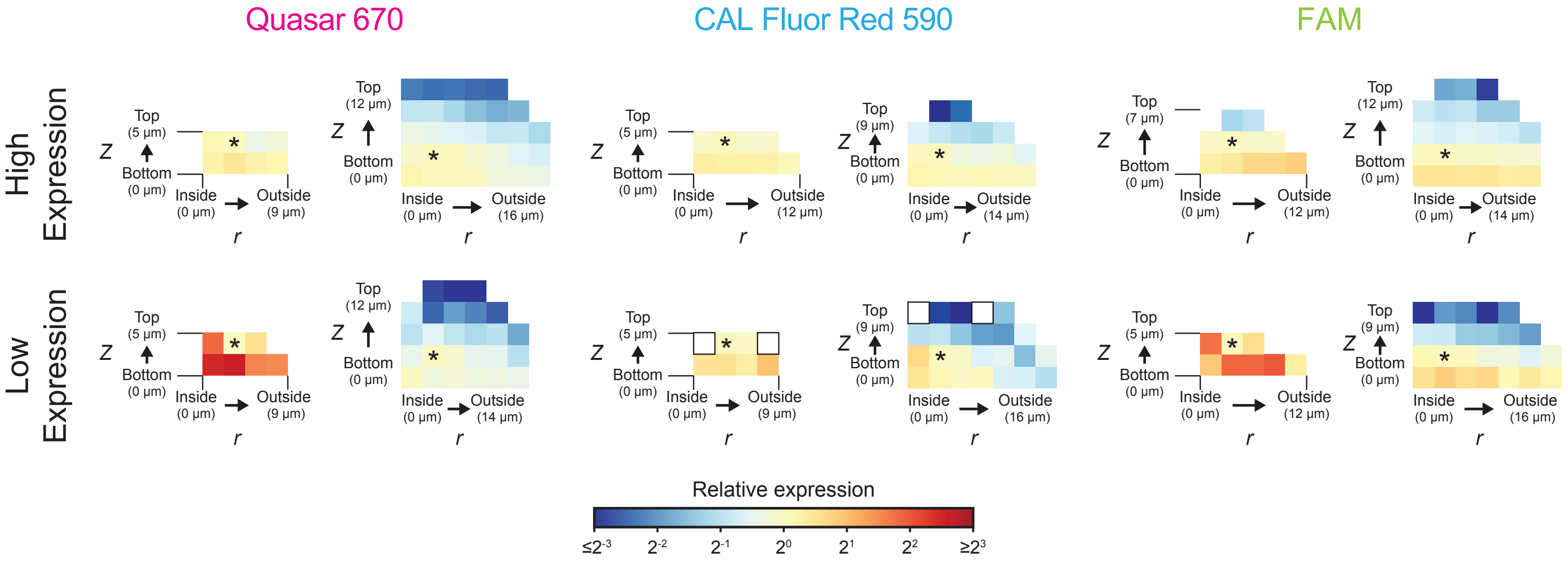

S3C, non-corrected data

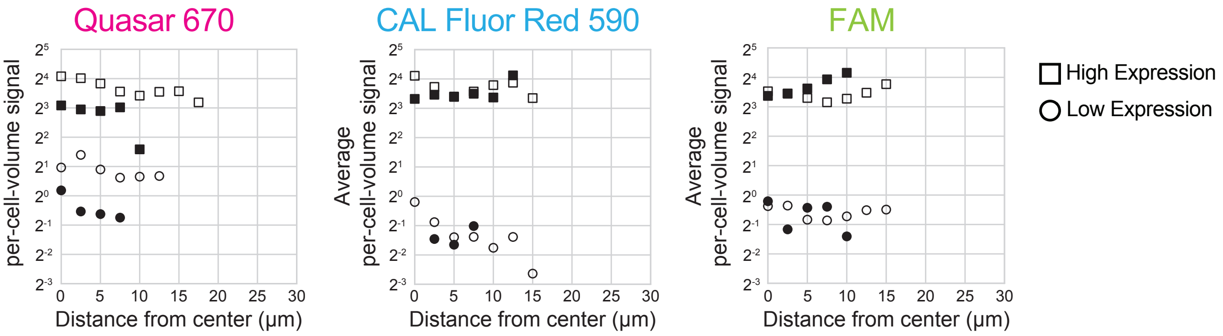

Fig 3A, non-corrected data

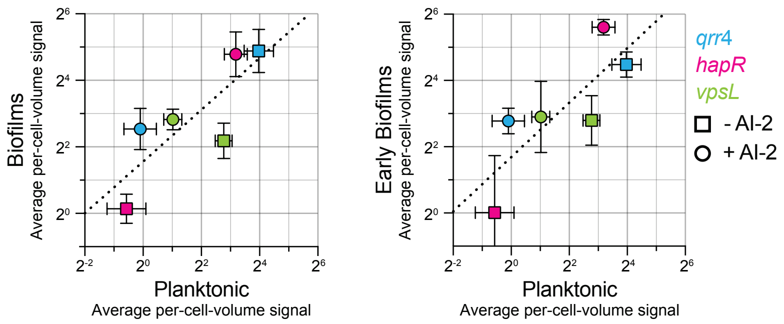

Fig 3B, non-corrected data

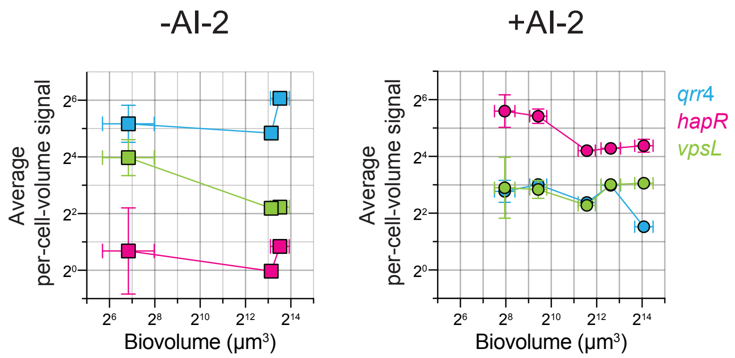

S6A, non-corrected data

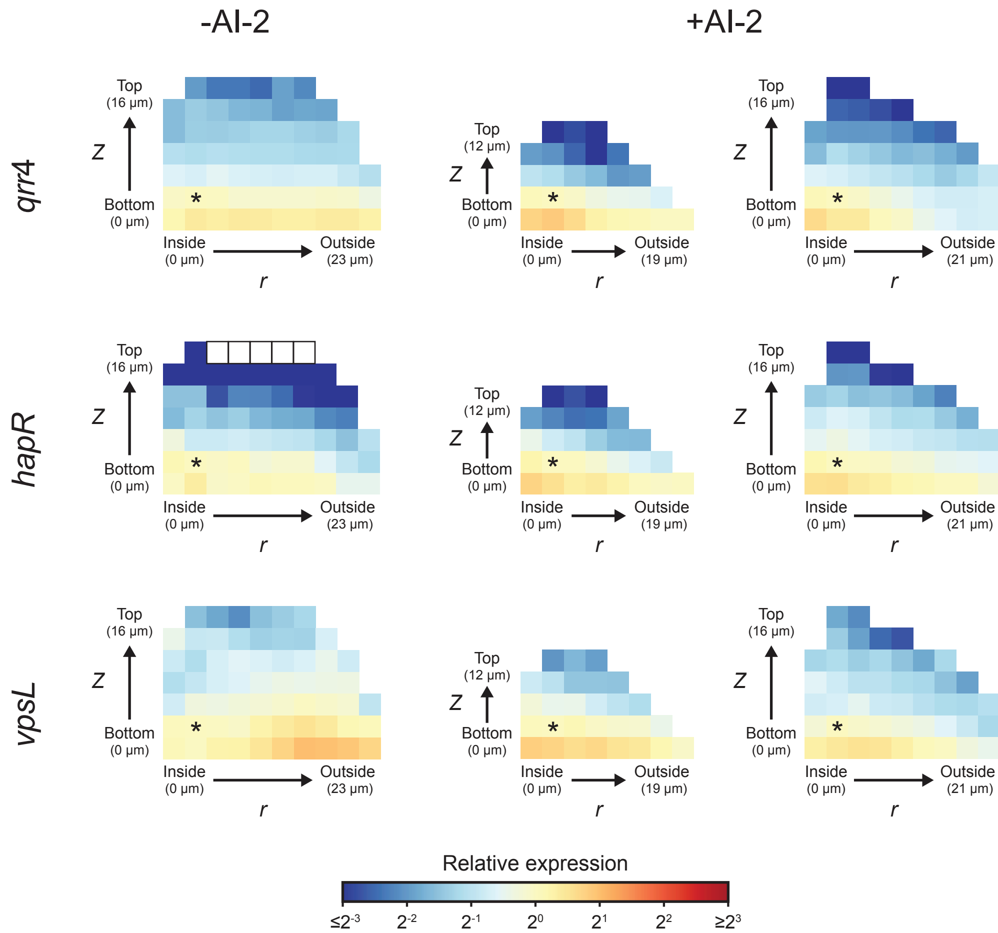

S6B, non-corrected data

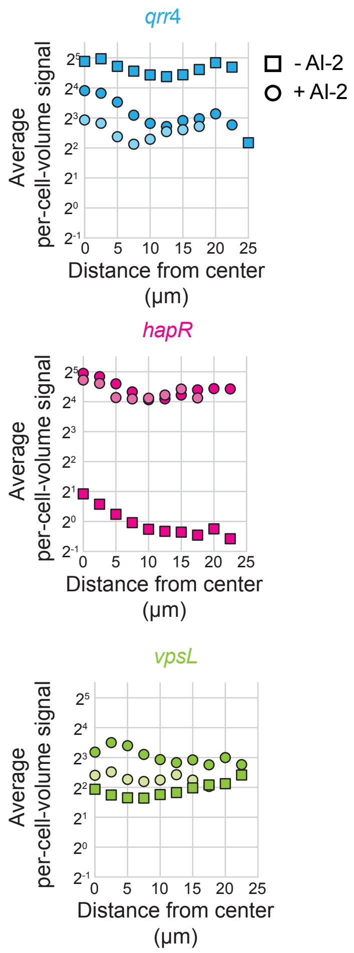

S7B, non-corrected data

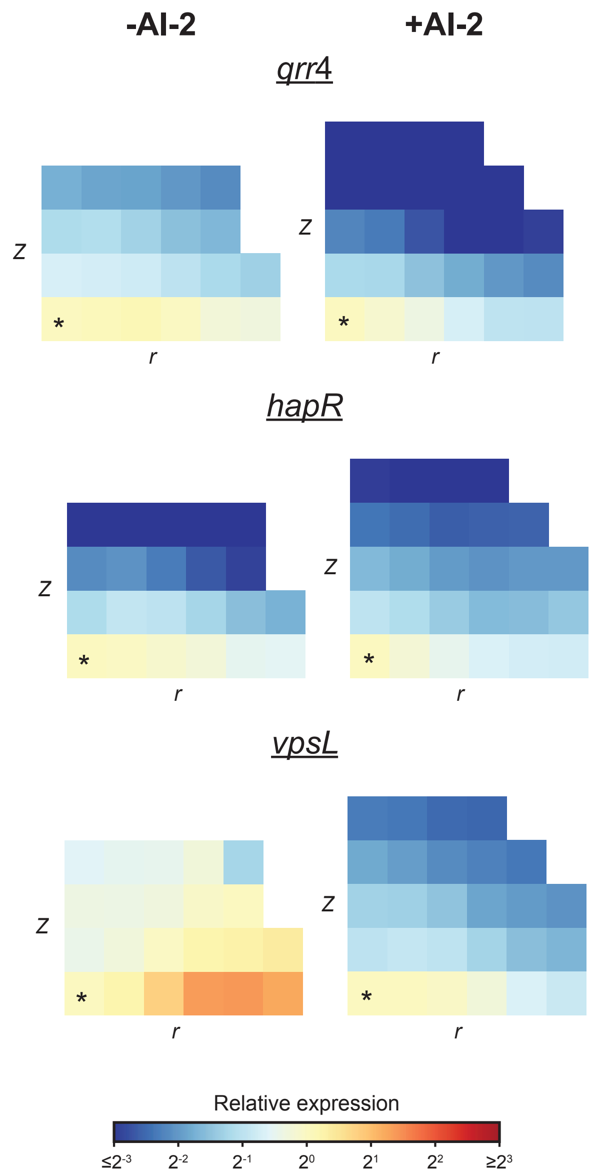

S7C, non-corrected data

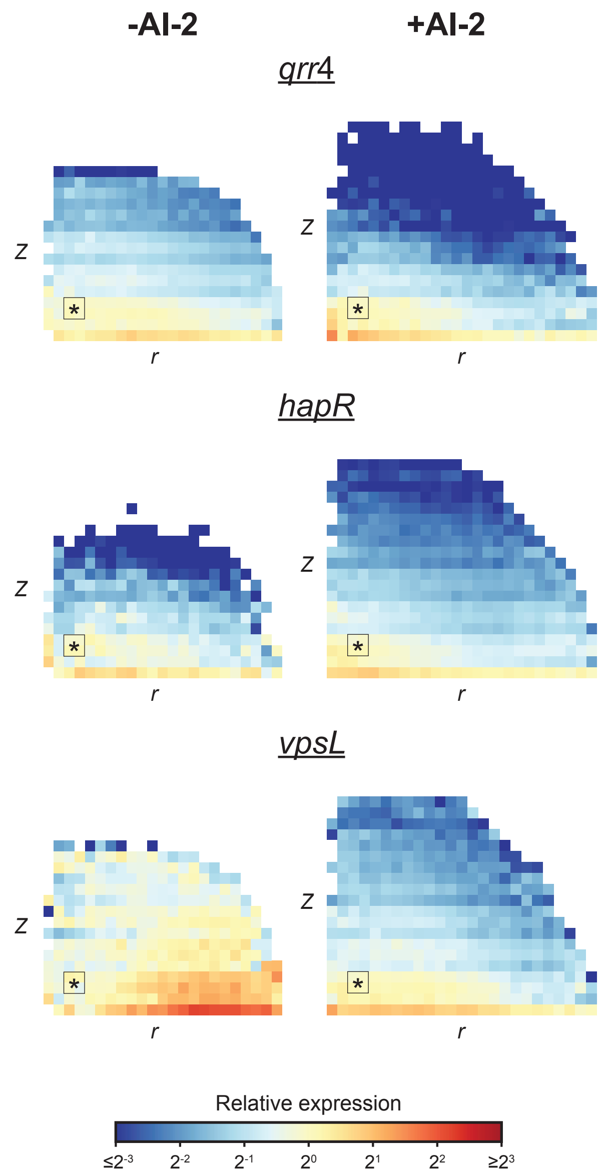

Fig 4A, non-corrected data

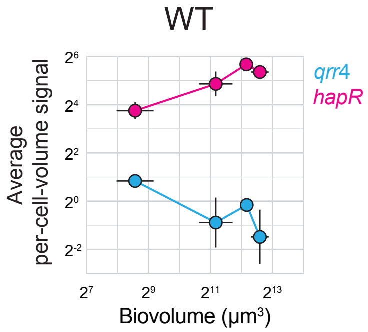

Fig 4B, non-corrected data

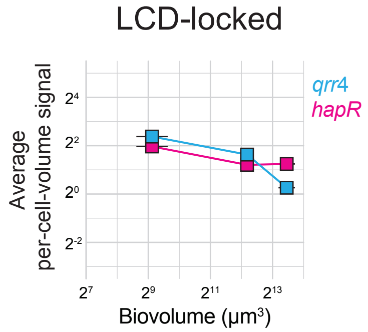

Fig 4C, non-corrected data

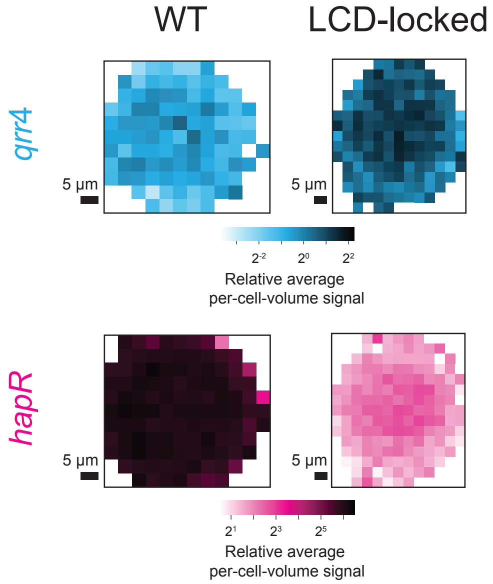

Fig 4D, non-corrected data

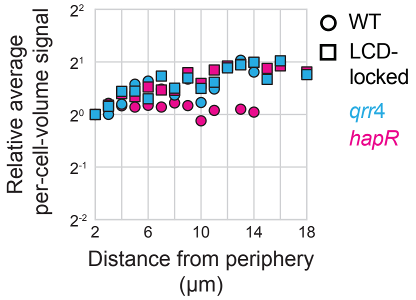

Fig 4E, non-corrected data

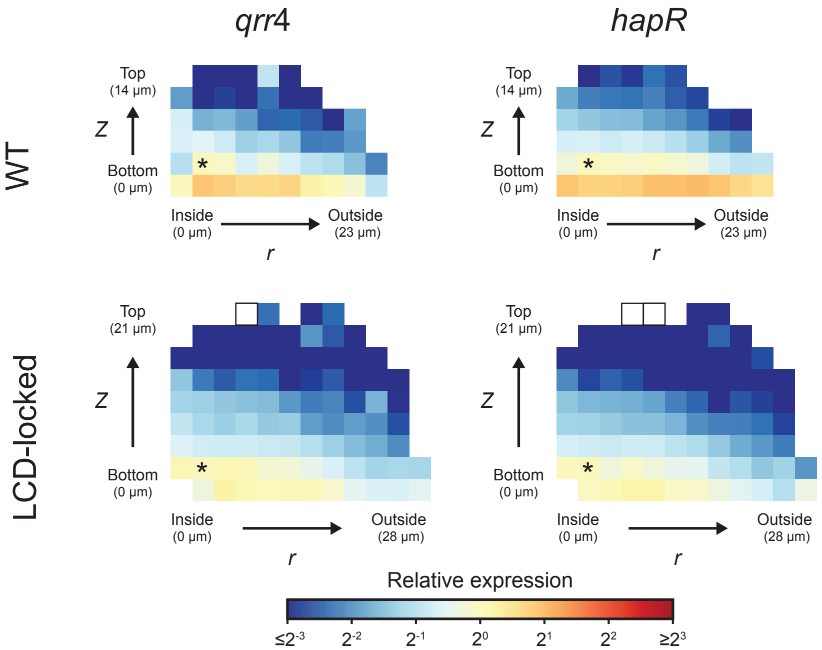

Fig 4F non-corrected data

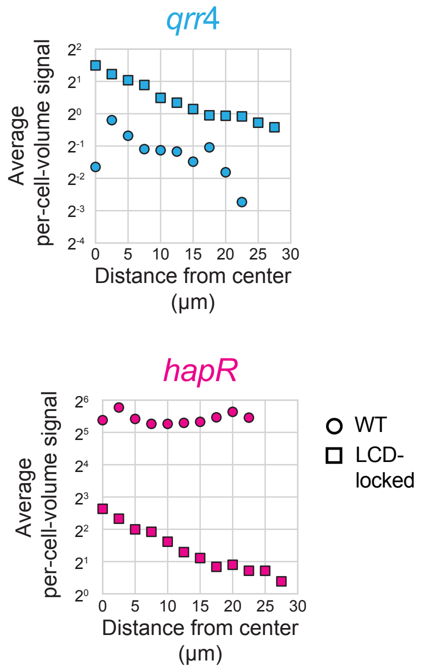

Fig 5A, non-corrected data

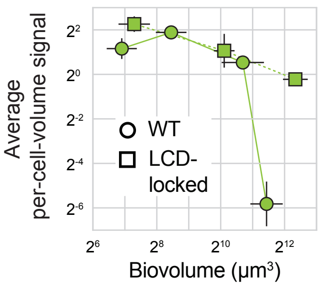

Fig 5B, non-corrected data

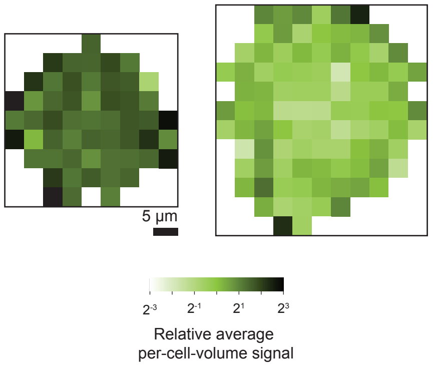

Fig 5C, non-corrected data

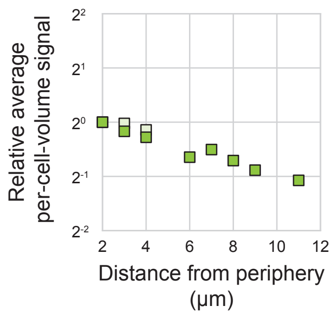

Fig 5D, non-corrected data

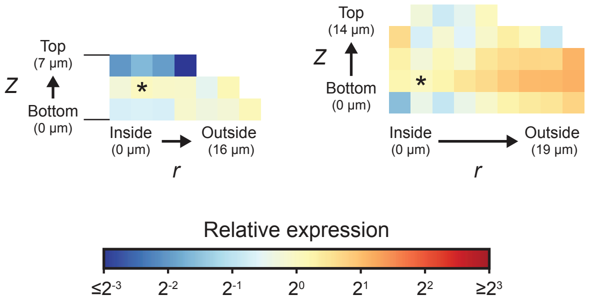

Fig 5E, non-corrected data

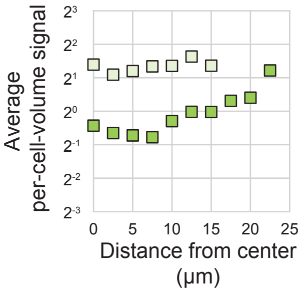

Fig 6A, non-corrected data

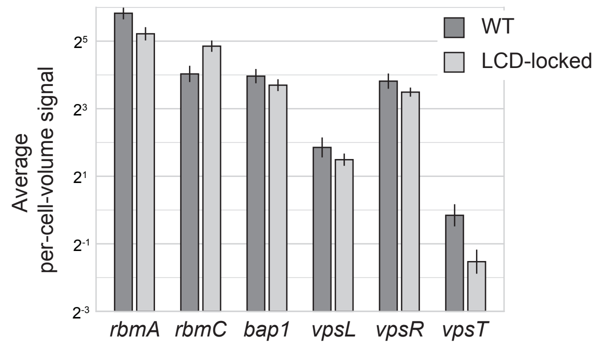

Fig 6B, non-corrected data

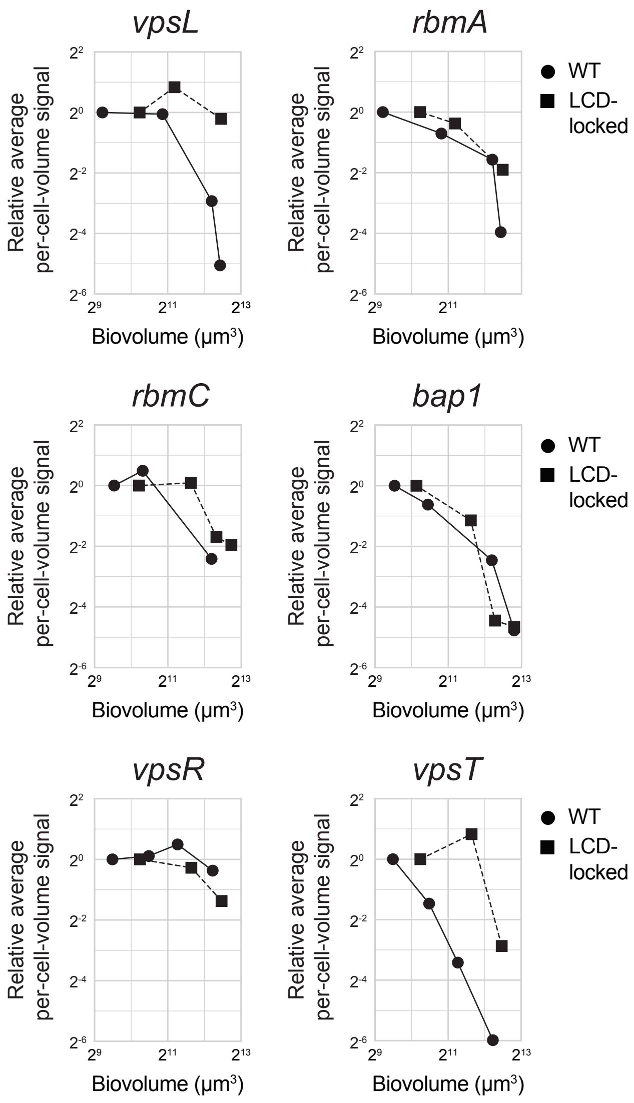

Fig 6C, non-corrected data

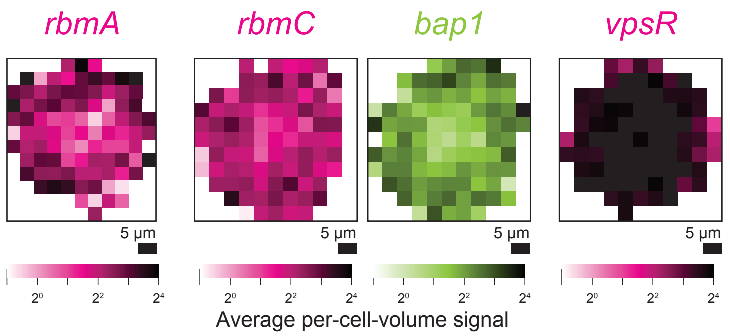

Fig 6D,
non-corrected data

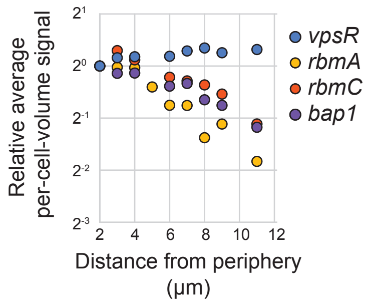

Fig 6E,
non-corrected data

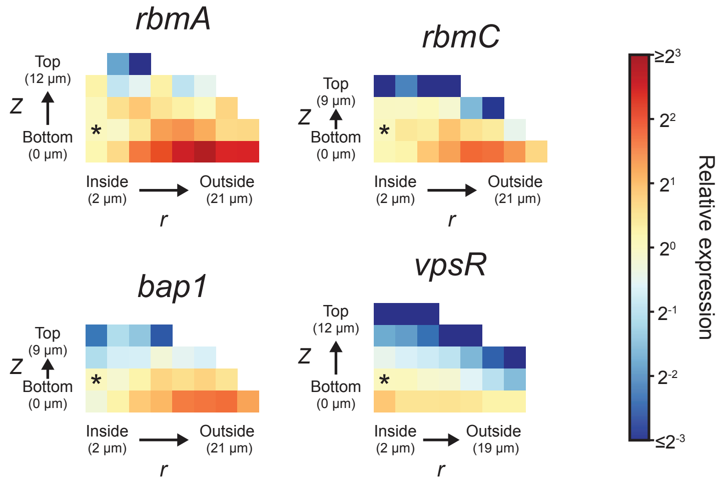

Fig 6F,
non-corrected data

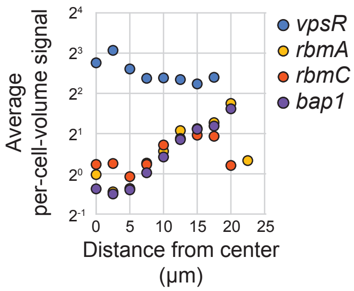

S9A, non-corrected data

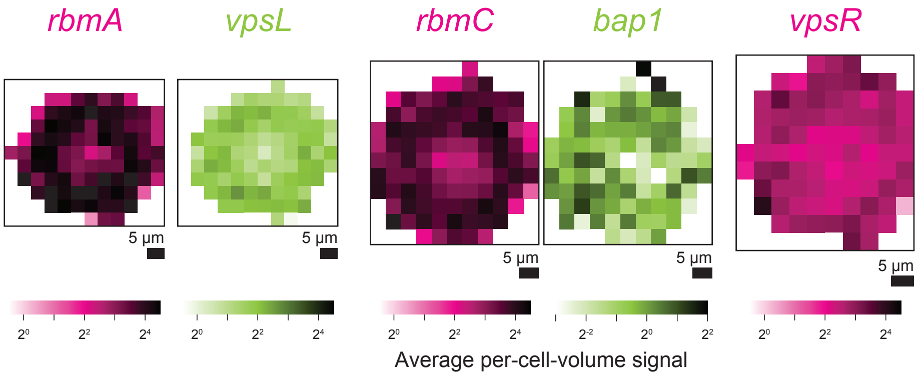

S9B, non-corrected data

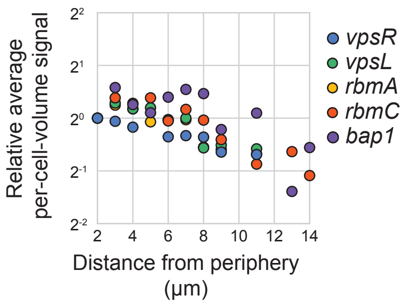

S9D, non-corrected data

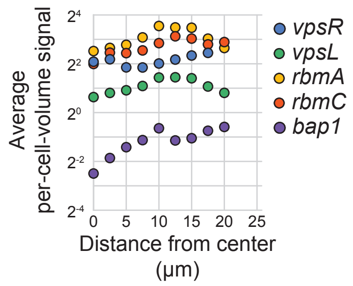

S9E, non-corrected data

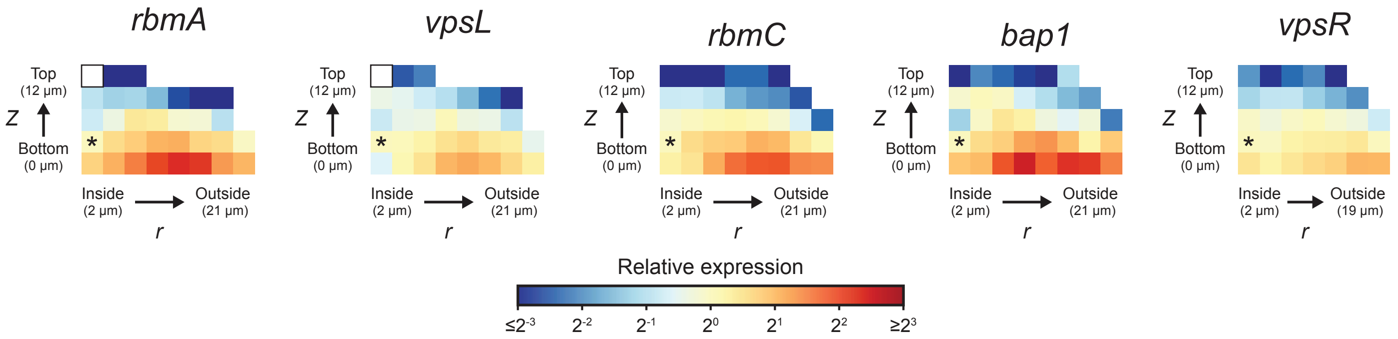

Supplement: S1 Data — For each plot, the corresponding main text or supplemental figure is indicated. (PDF) [file pbio.3003187.s005.pdf]
