# Supplementary material for: Analysis of gene expression within individual cells reveals spatiotemporal patterns underlying Vibrio cholerae biofilm development
Source: PLoS Biol. 2025 May 16;23(5):e3003187. doi: 10.1371/journal.pbio.3003187 (PMC12121927; doi:10.1371/journal.pbio.3003187)
Supplement: S2 Data — (PDF) [file pbio.3003187.s006.pdf]

Data S2. Validation of the linear interpolation of the spatial correction model.

To validate that the linear equations calculated from our model accurately describe the imaging bias across a range of signal values, we performed the following analysis: For each position (*r, z*), the linear equation used to calculate the correction factor from total biofilm signal for probes labeled with Quasar 670 was plotted (black curves). A representative plot, with (*r, z*) = 8, 8 μm, is highlighted here:

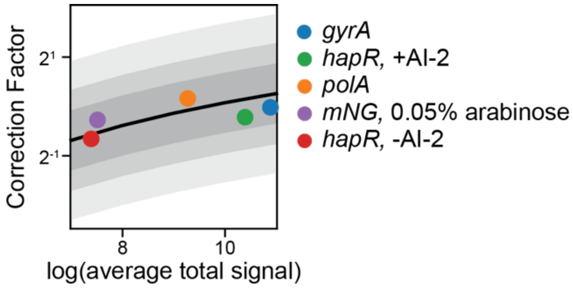

(*r, z*)-dependent correction factors were re-derived for smFISH datasets for genes targeted with Quasar 670 labeled probes for which expression is assumed to be uniform across the biofilm by fitting these data with Equation 1 and Equation 2 (see Methods: Spatial Correction Model). Calculated correction factors are plotted as a function of the average total smFISH fluorescent signal. The datasets included are: *gyrA* expression; *polA* expression; *hapR* expression in the AI-2 sensing strain grown without or with 10 μm AI-2; *mNG* expression in biofilms expressing *mNG* from the arabinose-inducible promoter and grown with 0.05% arabinose. Most points fall within 2-fold of the correction factor predicted from the linear interpolations, confirming the ability of these linear equations to generate correction factors that match signal bias at specific output values.

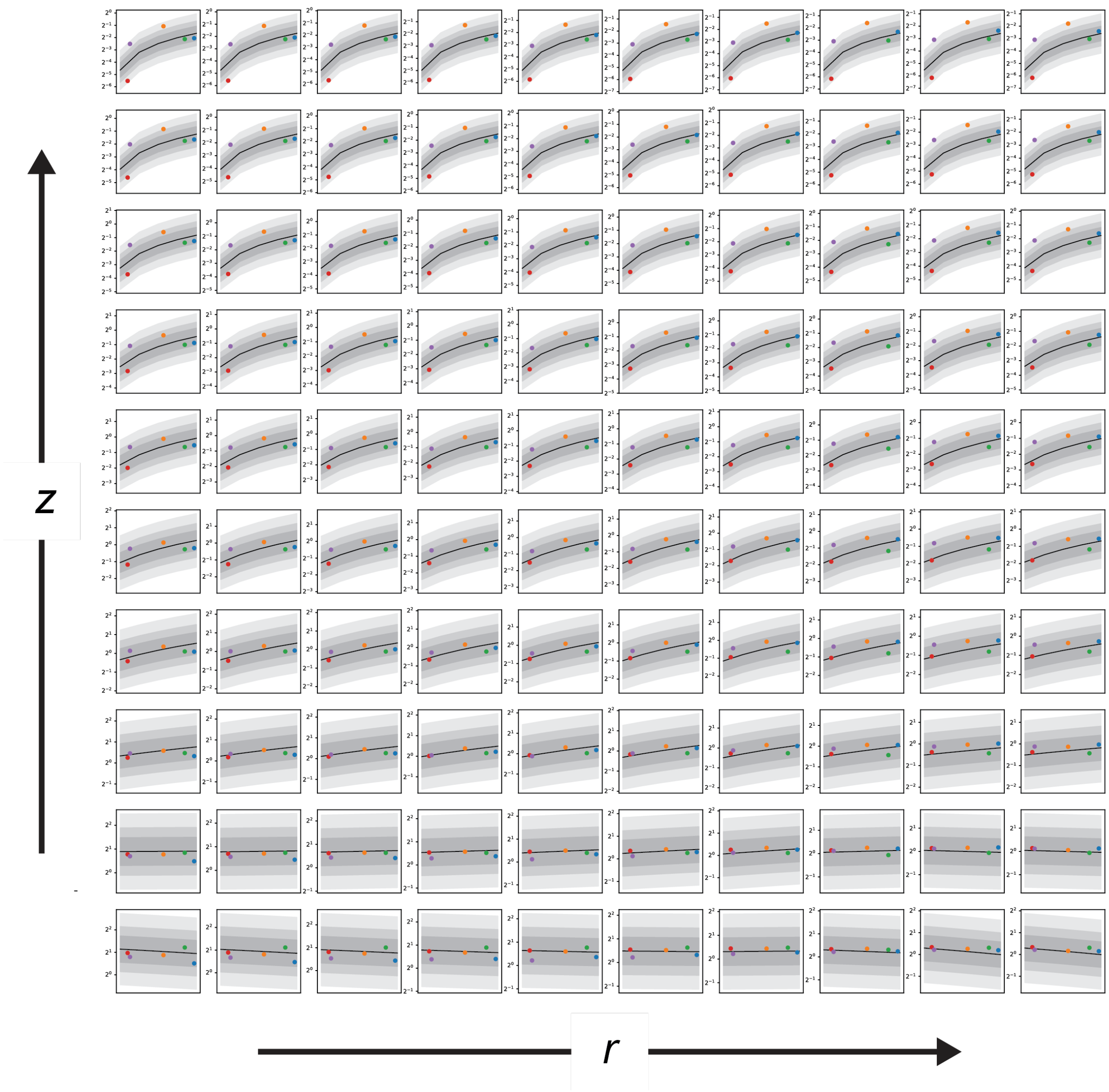

Visual representation of the validation of the linear interpolation of the spatial correction model. Black curves in plots: linear equations Y = mX+b used to correct data, where X is the log of the average summed smFISH fluorescence signals across replicate biofilms and Y is the (*r, z*)-specific correction factor for those biofilms. A $\log_2$ Y axis is used, so the linear fit appears curved. Color points: correction factors calculated using *gyrA* (blue), *polA* (orange), *hapR* (red, - AI-2, green, +AI-2), or *mNG* (purple) expression. Shading of dark gray, medium gray, and light gray represents 1.5, 2, and 3-fold deviations from the linear interpolation. Each graph shares the same X range as the one highlighted above, and shows a unique position *(r, z*) ranging from 0-23 μm.
