# Supplementary figures and images for: Analysis of gene expression within individual cells reveals spatiotemporal patterns underlying Vibrio cholerae biofilm development
Source: PLoS Biol. 2025 May 16;23(5):e3003187. doi: 10.1371/journal.pbio.3003187 (PMC12121927; doi:10.1371/journal.pbio.3003187)

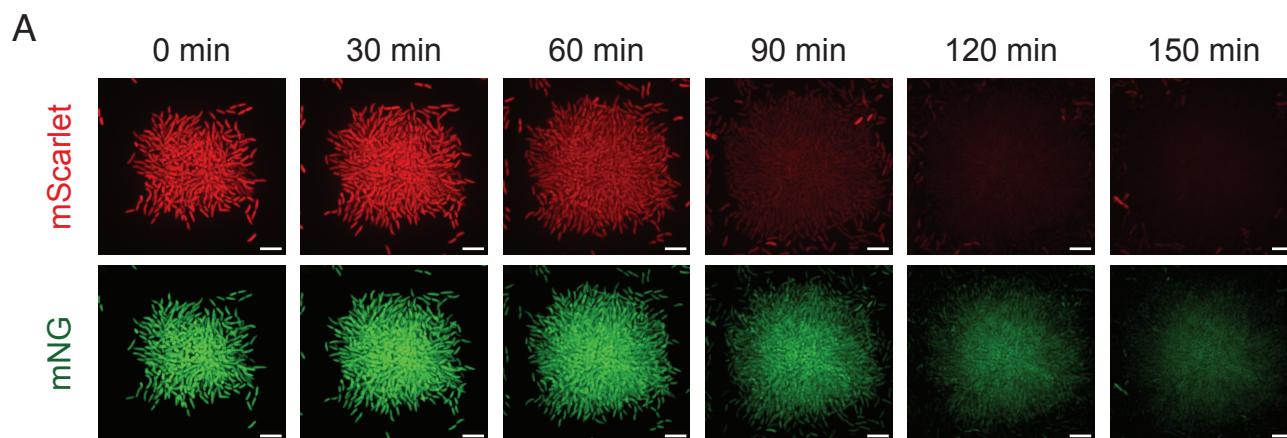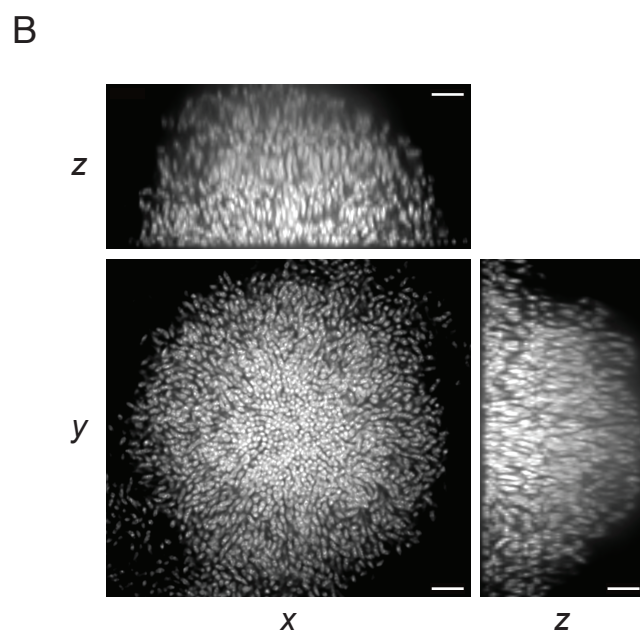

Supplement: S1 Fig — (A) Maximum projection confocal microscopy images of mScarlet and mNG fluorescence over time in a representative V. cholerae biofilm harboring pTac-mScarlet and pTac-mNG. (B) Signal from DAPI staining in the first in-focus z slice and xz and yz cross sections of a mature V. cholerae biofilm. All scale bars represent 5 µm. (PDF) [file pbio.3003187.s007.pdf]

A

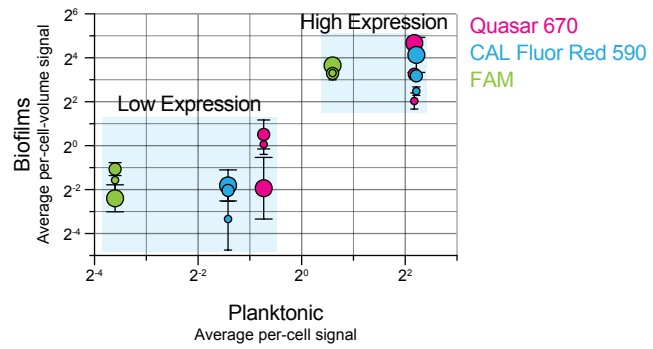

D

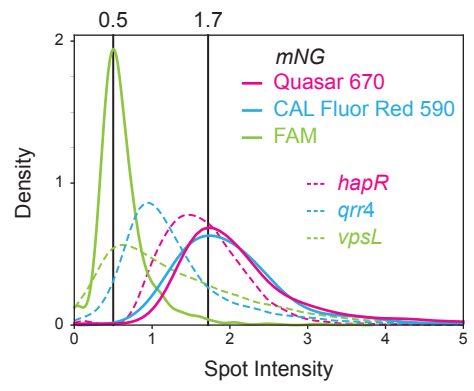

B

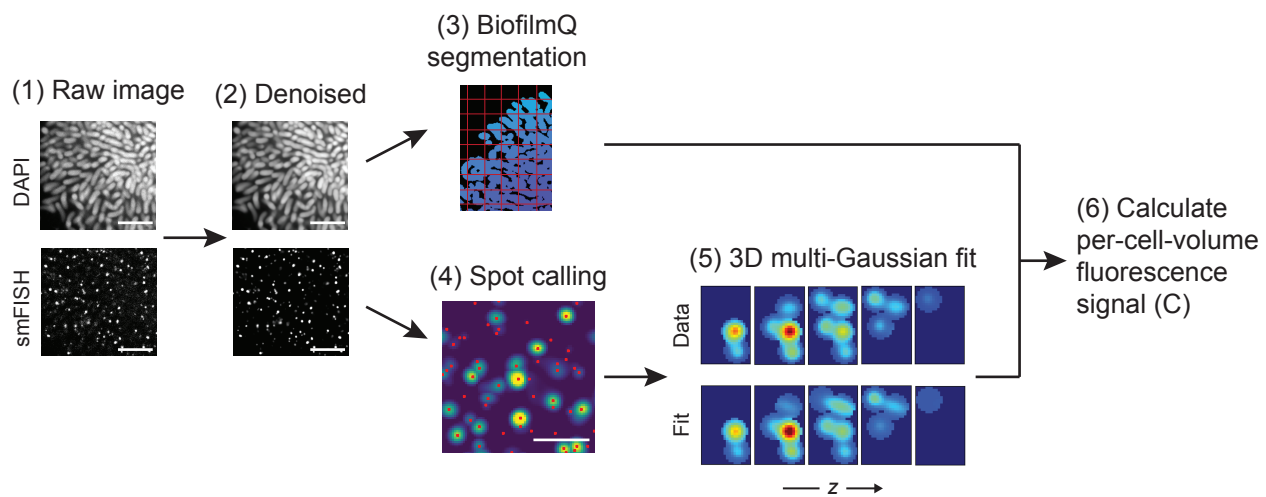

C

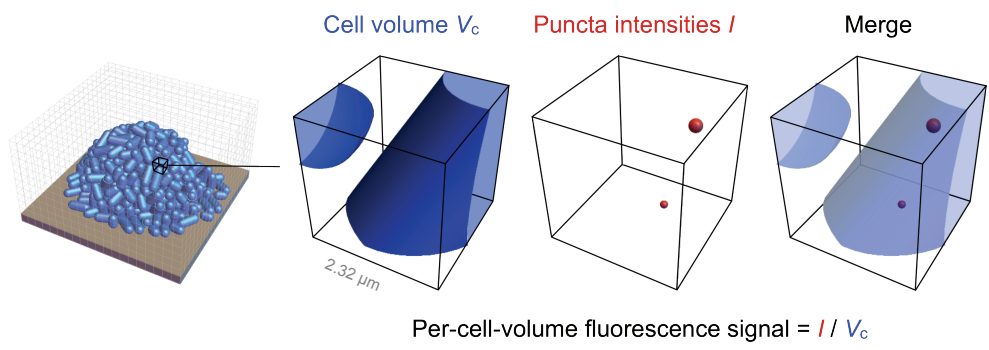

E

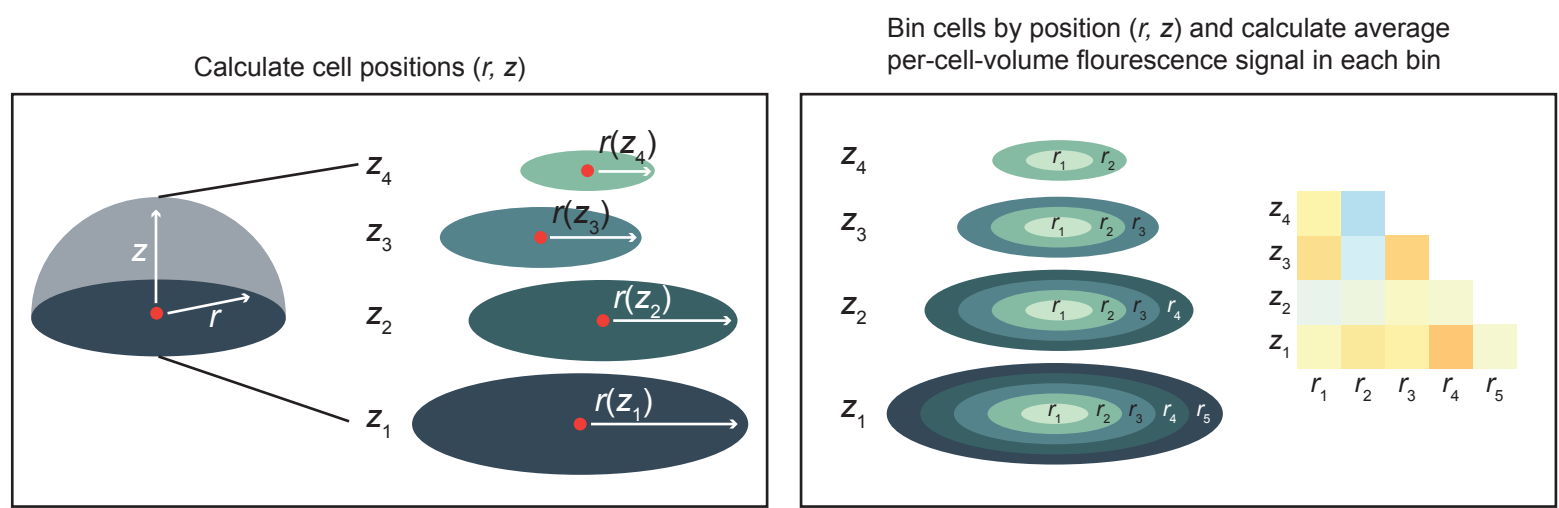

Supplement: S2 Fig — (A) Average per-cell-volume mNG smFISH fluorescence signal measured in V. cholerae planktonic and biofilm cells. Arabinose was provided at concentrations of 0.2% or 0.0375%, as indicated by the shaded blue boxes labeled High and Low Expression, respectively. In all cases, 100 µM Nspd was included. mNG expression was measured by smFISH using probes labeled with one of three fluorophores, as indicated. Values for biofilm cells represent the mean normalized per-cell-volume fluorescence signal, calculated as described in (B), across n = 10–12 biofilms. The small, medium, and large circular symbols represent biofilms with approximate biovolumes of 29, 211, and 214 µm3, respectively. Error bars denote standard deviations, which are in some cases smaller than the sizes of the symbols used in the plots. Values for planktonic cells represent the average across all cells in single replicate experiments; error bars are excluded. (B) Schematic overview of smFISH image analysis. Briefly, (1) raw images are processed with NIS-Elements Denoise.ai software to (2) generate denoised images. Image scale bars in (1) and (2) represent 5 µm. (3) Using the DAPI channel, BiofilmQ [43] is used to segment biofilms into cubes (outlined in red) with side lengths of 2.32 µm and cells are identified within these cubes (highlighted in blue). (4) Puncta from smFISH fluorescence signal are detected as local maxima. In (4) scale bar represents 1 µm and red dots represent called smFISH puncta. (5) Puncta are fitted with a 3D Gaussian function to calculate the integrated punctum intensity. (6) Per-cell-volume fluorescence signal is calculated as described in (C). (C) Schematic overview of quantitation of per-cell-volume fluorescence signal. Briefly, the BiofilmQ segmentation is used to calculate cell volume (Vc) for each cube, defined as the fraction of the total cube volume occupied by cell mass. smFISH fluorescence signal is used to calculate punctum intensity (I). The per-cell-volume fluorescence s [file pbio.3003187.s008.pdf]

A

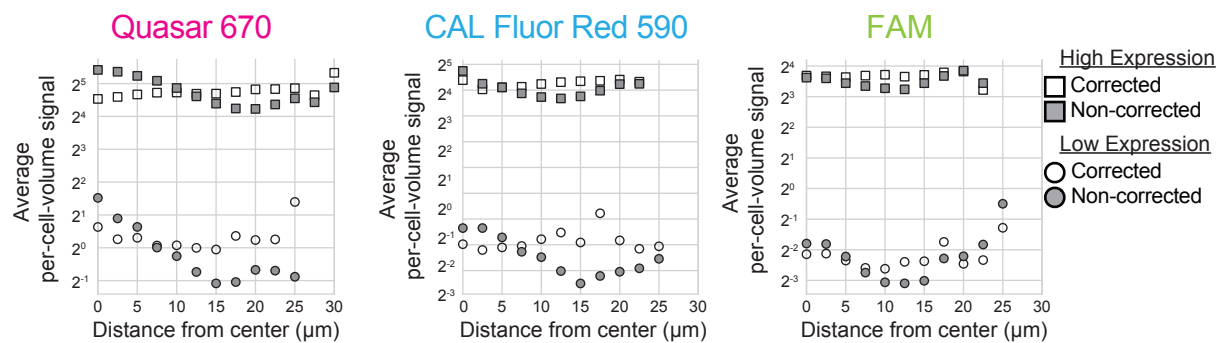

B

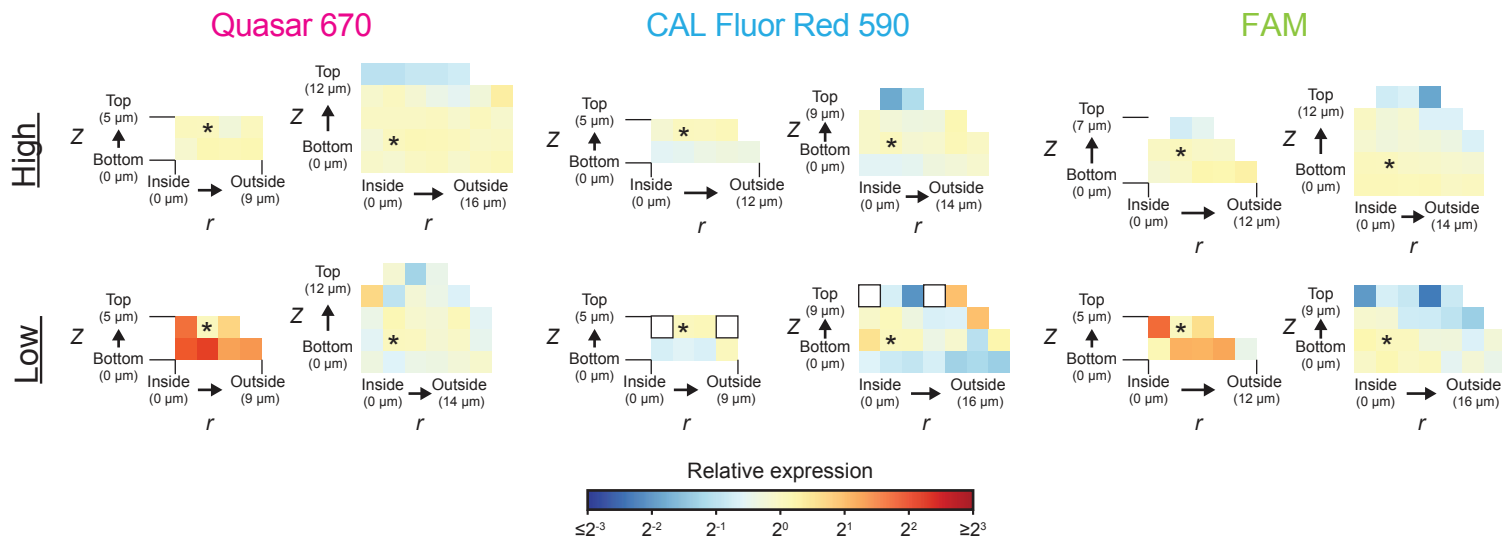

C

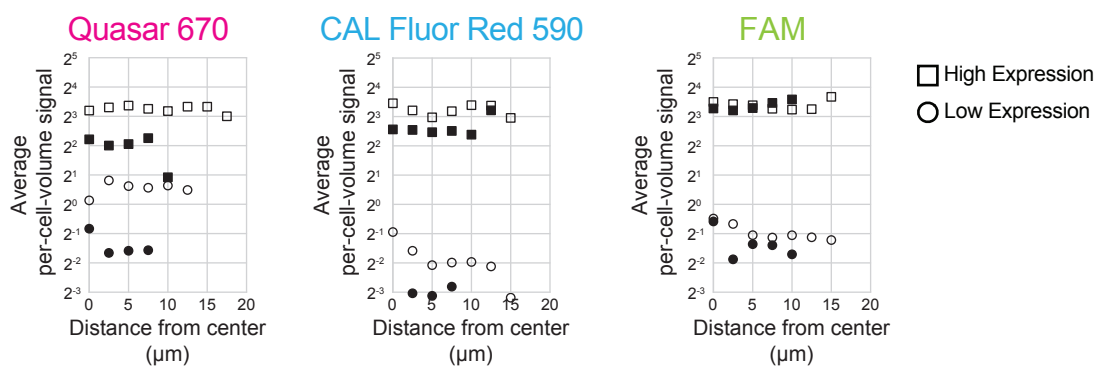

Supplement: S3 Fig — (A) Average per-cell-volume fluorescence signal versus distance to the biofilm center (r, z = 0, 3.5 µm) is shown for each fluorophore as indicated. The corrected and non-corrected data are shown for biofilms with high and low mNG expression. All data represent n = 10–12 biofilms. (B) Heatmaps as in Fig 2A of the main text showing the relative corrected data for the three fluorophores for biofilms of additional sizes. Data represent n = 10–12 replicate biofilms. (C) As in (A), for the data shown in (B). Black symbols represent biofilms in the left column (smaller biofilms) and white symbols represent biofilms in the right column (larger biofilms) of (B). The data underlying this figure can be found in S1 Table. (PDF) [file pbio.3003187.s009.pdf]

A

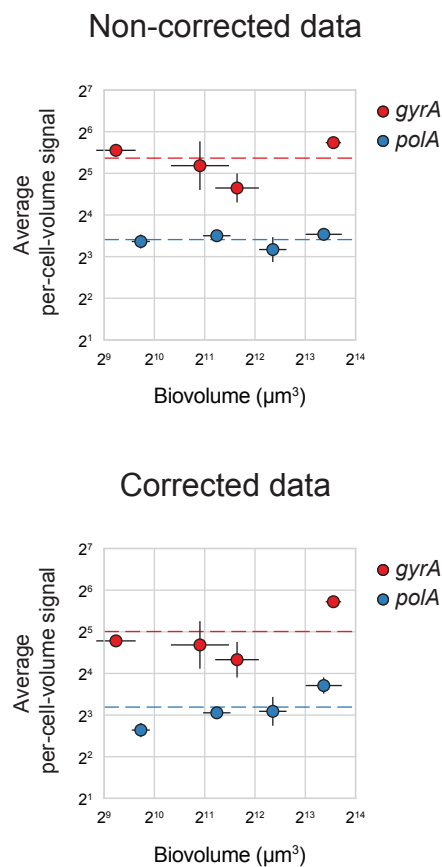

B

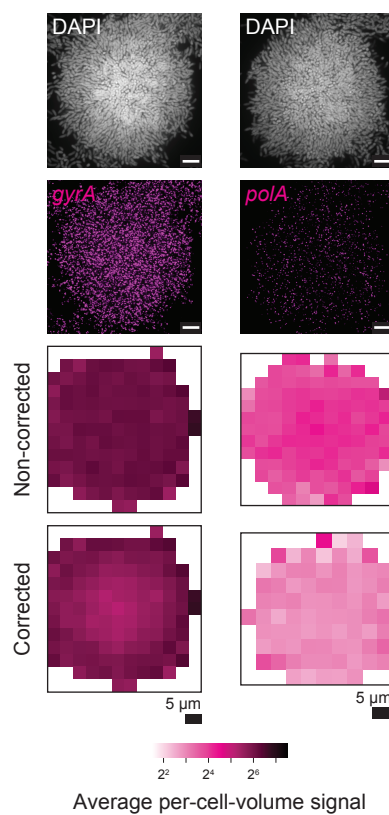

C

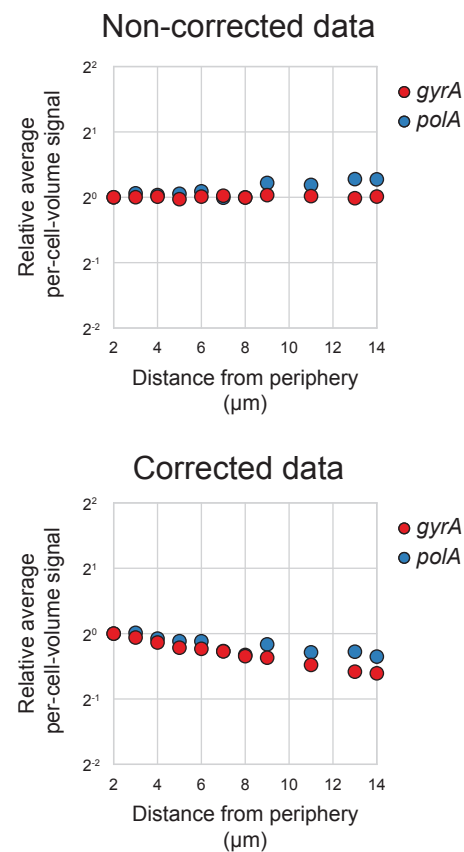

D

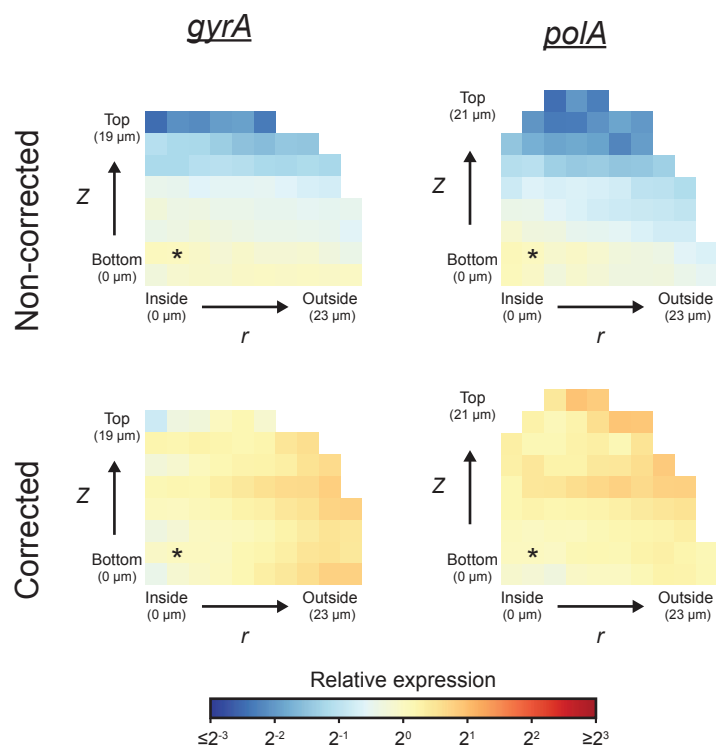

E

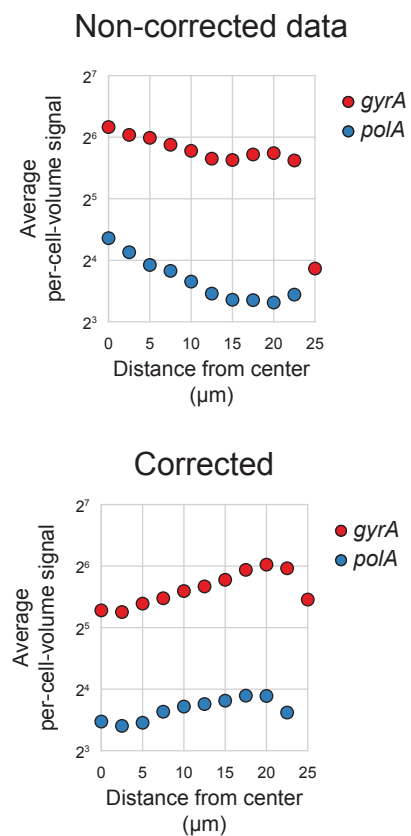

Supplement: S4 Fig — (A) Average per-cell-volume gyrA and polA smFISH fluorescence signal before (top) and after (bottom) correction using the model described in the text (See Methods: Spatial correction model) across replicate V. cholerae biofilms grown with 100 µM Nspd as a function of the average biofilm biovolume. Dotted lines denote the average smFISH fluorescence signal across all biovolumes. Error bars denote standard deviations across n = 9–10 replicate biofilms, which are in some cases smaller than the sizes of the symbols used in the plots. (B) (Top) Representative confocal microscopy images showing DAPI, and gyrA and polA smFISH fluorescence signal in biofilms with the largest biovolumes shown in (A). Images represent maximum projections of the first four in-focus z slices. Scale bars represent 5 µm. (Bottom) Heatmaps showing average gyrA and polA non-corrected (upper) and corrected (lower) per-cell-volume smFISH fluorescence signal across n = 10 replicate biofilms as a function of x, y position (with replicate biofilms aligned around their center positions) for biofilm cells with z positions between 0 and 4.68 µm in biofilms with the largest biovolumes shown in (A). Scale bars represent 5 µm. (C) Non-corrected (top) and corrected (bottom) relative average per-cell-volume fluorescence signal as a function of distance from the periphery of the biofilm for the data shown in (B). Values are relative to the average per-cell-volume fluorescence signal of cells located at a distance of 2 µm from the biofilm periphery for each gene. (D) Heatmaps of gyrA and polA non-corrected (top) and corrected (bottom) average per-cell-volume fluorescence signal relative to the average per-cell-volume fluorescence signal in the bins denoted with asterisks are shown as in Fig 2B for n = 10 replicate biofilms with the largest biovolumes shown in (A). (E) Average non-corrected (top) and corrected (bottom) per-cell-volume fluorescence signal versus distance to the biofilm center, as in S3A Fig, for th [file pbio.3003187.s010.pdf]

A

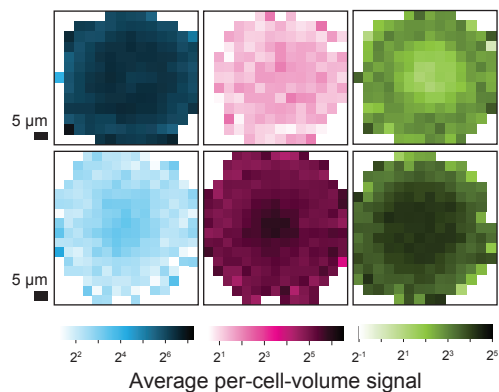

B

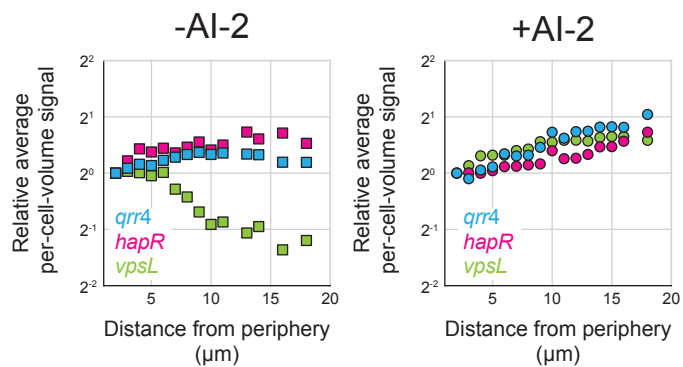

C

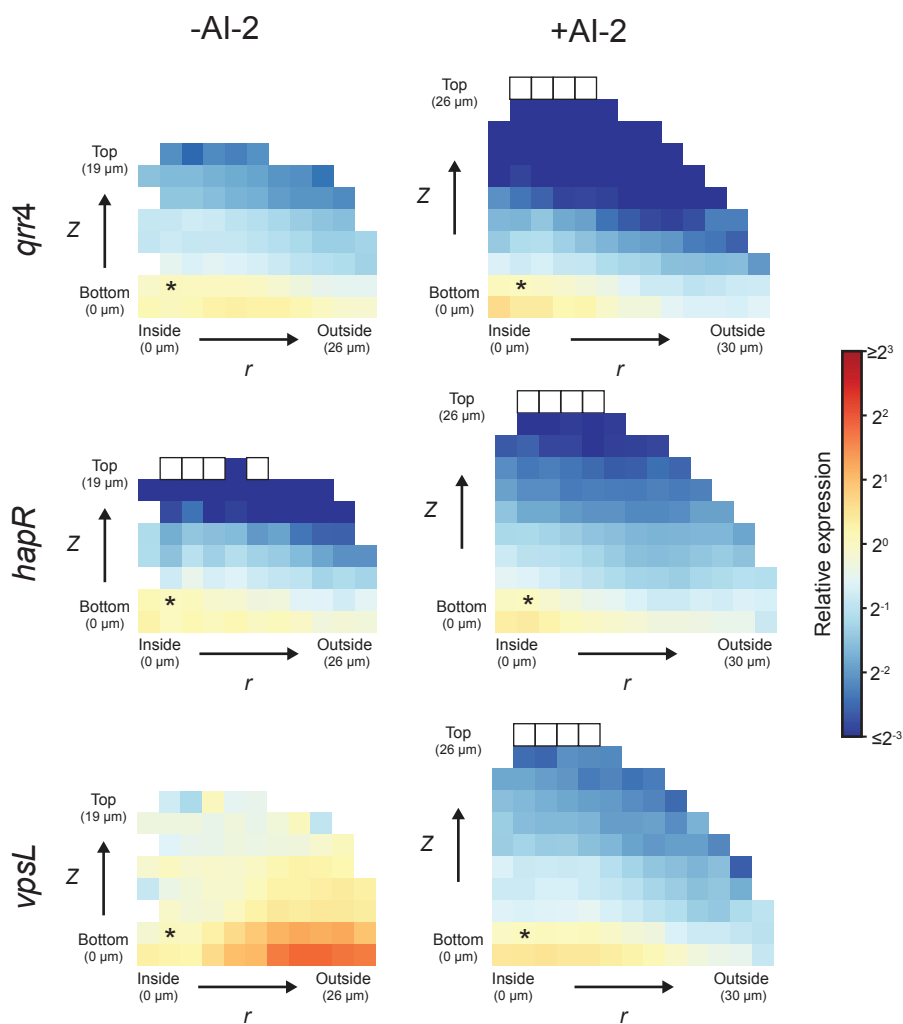

D

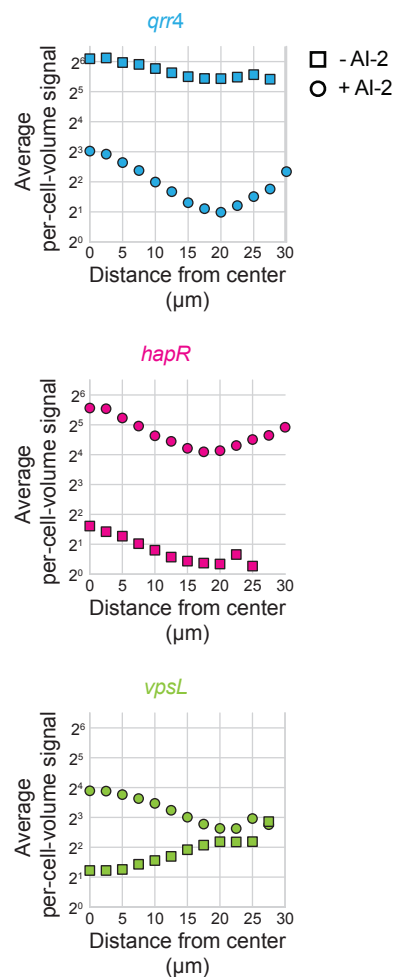

E

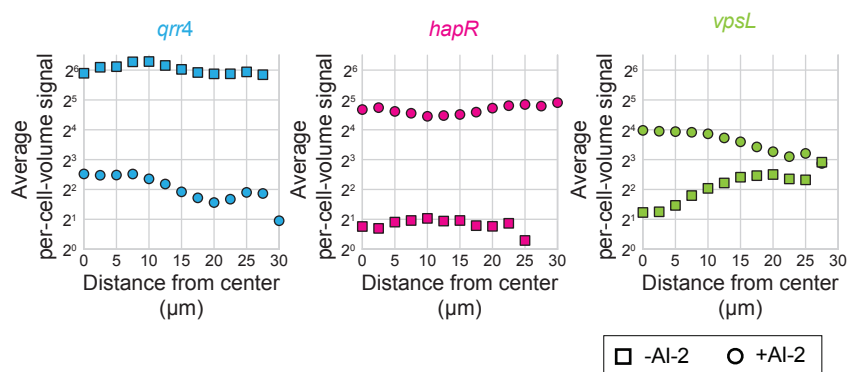

Supplement: S5 Fig — (A, B, C, D) The non-corrected data used to generate the spatially corrected fluorescence signal values shown in Figs 3C–3E, and S5E, respectively. (E) Average per-cell-volume fluorescence signal versus distance to the biofilm center, as in S3A Fig, for the data shown in Fig 3E. The data underlying this figure can be found in S1 Table. (PDF) [file pbio.3003187.s011.pdf]

B

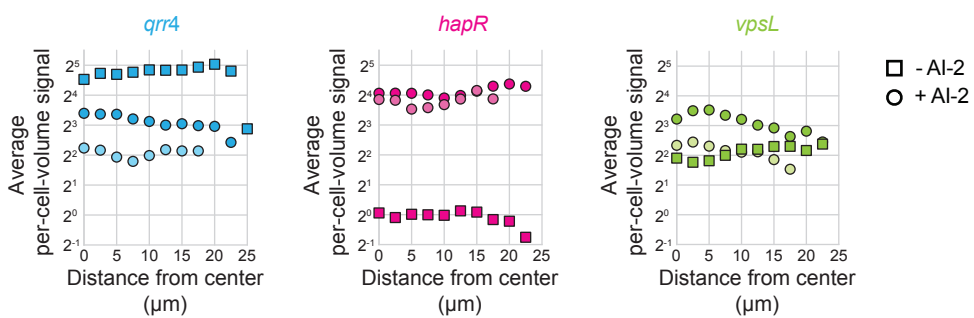

Supplement: S6 Fig — (A) Heatmaps showing the relative qrr4 (mNG), hapR, and vpsL per-cell-volume fluorescence signals as in Fig 2B of the main text for additional biofilm sizes. n = 9−10 replicate biofilms. (B) Average per-cell-volume fluorescence signal versus distance to the biofilm center is shown, as in S3A Fig, for the data represented in (A). Light symbol coloring for +AI-2 samples represent the smaller biofilms (left in (A)) and dark symbol coloring for +AI-2 samples represent larger biofilms (right in (A)). All data have been corrected using the model described in the text (See Methods: Spatial correction model). The data underlying this figure can be found in S1 Table. (PDF) [file pbio.3003187.s012.pdf]

A

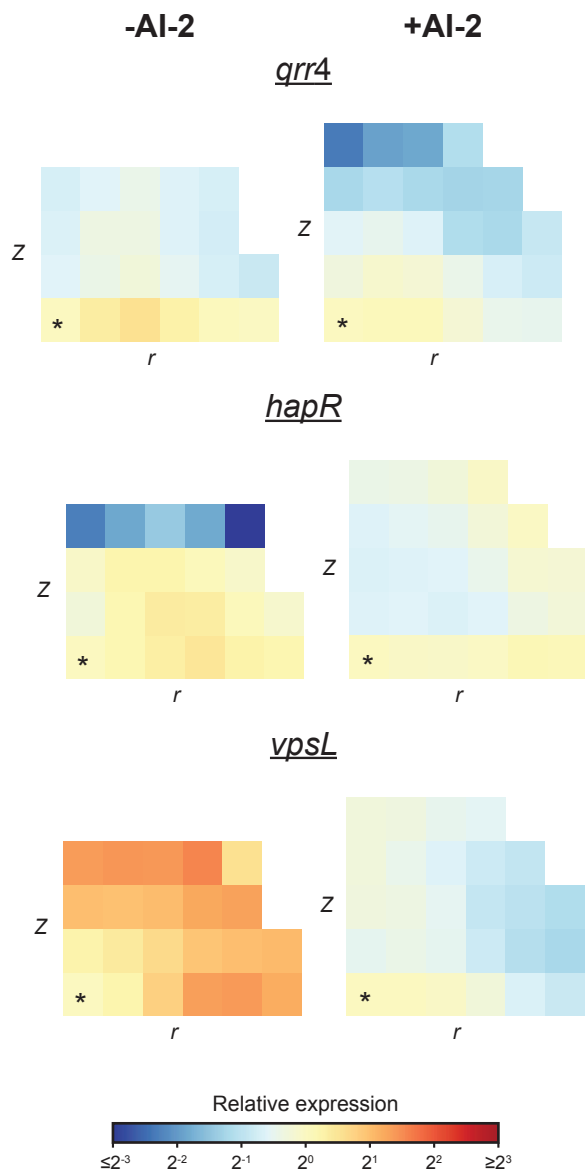

B

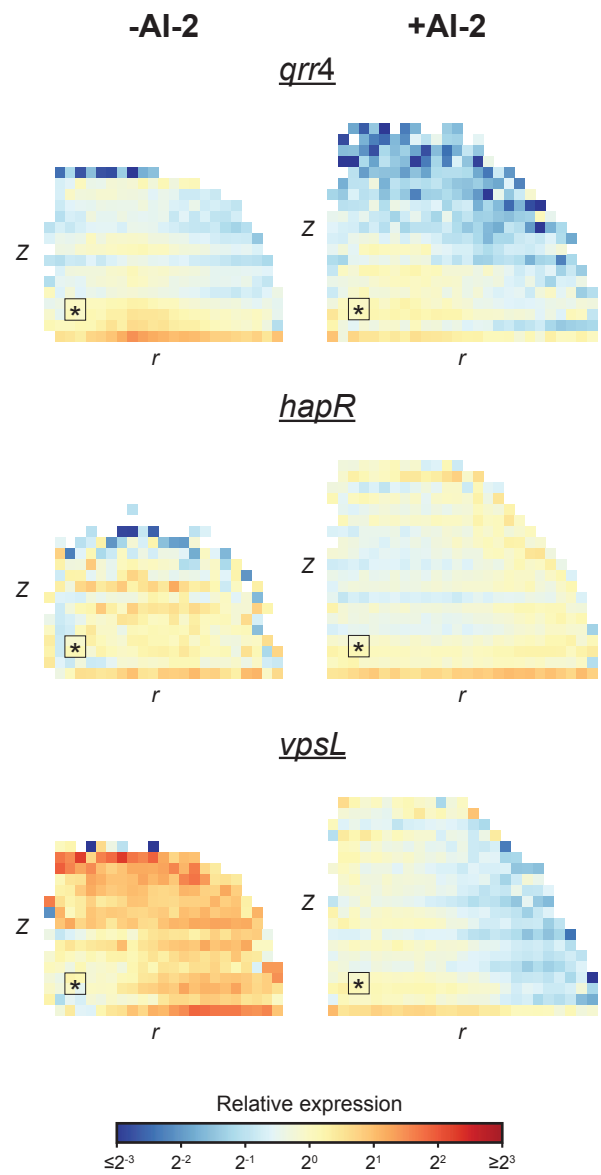

Supplement: S7 Fig — (A) Heatmaps showing the relative qrr4 (mNG), hapR, and vpsL per-cell-volume fluorescence signals for the data in Fig 3E, with biofilms segmented and cells binned using a cube side length of 4.64 µm. The per-cell-volume fluorescence signal is relative to the average per-cell-volume signal in the bin marked with an asterisk. (B) As in (A) with a cube side length of 1.15 µm. The per-cell-volume fluorescence signal is relative to the average per-cell-volume signal across bins outlined and marked with an asterisk. n = 10 replicate biofilms. The data underlying this figure can be found in S1 Table. (PDF) [file pbio.3003187.s013.pdf]

A

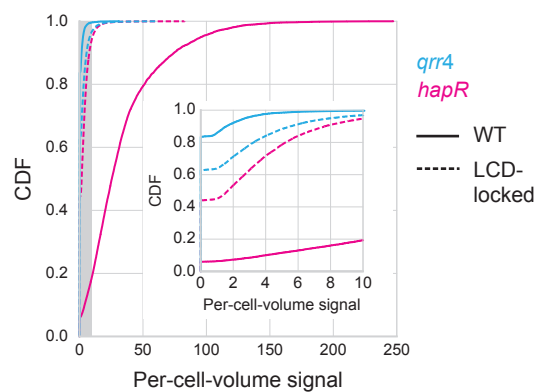

B

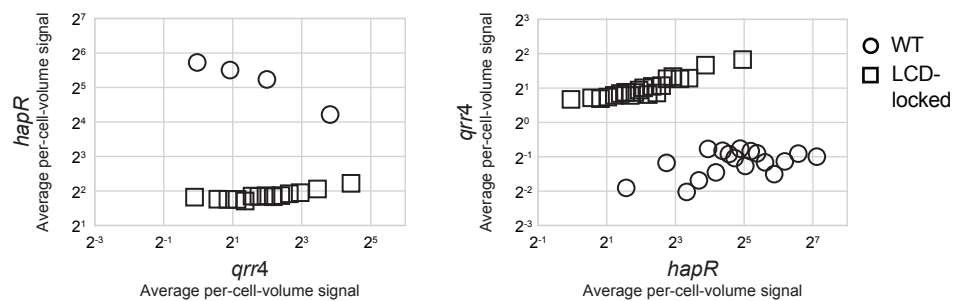

C

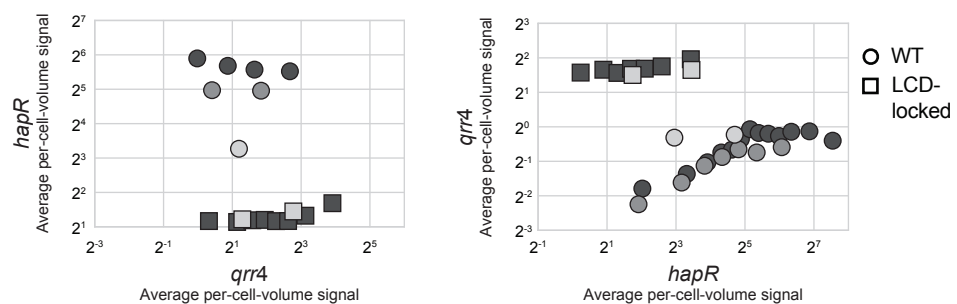

D

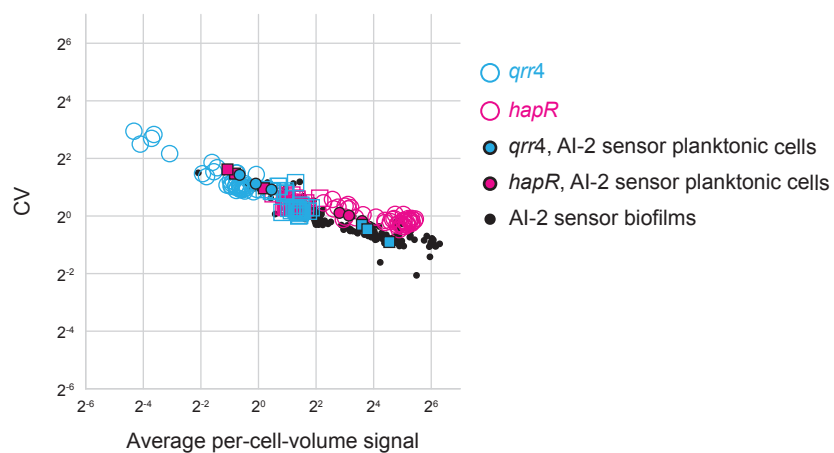

Supplement: S8 Fig — (A) Cumulative distributions of per-cell-volume qrr4 (mNG) and hapR fluorescence signals across individual cells in WT biofilms and biofilms of the LCD-locked strain shown in Fig 4C–4F. The inlay shows the region of the distribution highlighted in gray. (B) (Left) Average qrr4 (mNG) per-cell-volume fluorescence signal as a function of hapR per-cell-volume fluorescence signal for groups of cells in WT biofilms and biofilms of the LCD-locked strain shown in Fig 4C–4F. Values calculated as described in Methods: Correlating gene expression in cell groups, using qrr4 as the reference gene. (Right) As on the left, with hapR as the reference gene. (C) As in (B) for additional biofilm sizes. Dark to light shading represents biofilms of decreasing size. (D) The relationship between the coefficient of variation (CV) for qrr4 (mNG) or for hapR per-cell-volume fluorescence signal across cells in individual biofilm or planktonic samples, as indicated, as a function of the average qrr4 (mNG) or hapR per-cell-volume signal in the sample. Empty symbols represent biofilms of the WT (circles) or of the LCD-locked strain (squares) (data from Fig 4). Slopes of the best fit lines for qrr4 and hapR for these samples are −0.42 and −0.17, respectively. Filled colored symbols represent planktonic samples of the AI-2 sensor strain grown with (circles) or without (squares) AI-2 (planktonic data from Fig 3). Slopes of the best fit lines for qrr4 and hapR for these samples are −0.43 and −0.37, respectively. Black points represent both qrr4 (mNG) and hapR expression in biofilms of the AI-2 sensor strain grown with or without AI-2 (biofilm data from Fig 3). Slopes of the best fit lines for qrr4 and hapR for these samples are −0.28 and −0.37, respectively. All data have been corrected using the model described in the text (See Methods: Spatial correction model). The data underlying this figure can be found in S1 Table. (PDF) [file pbio.3003187.s014.pdf]

A

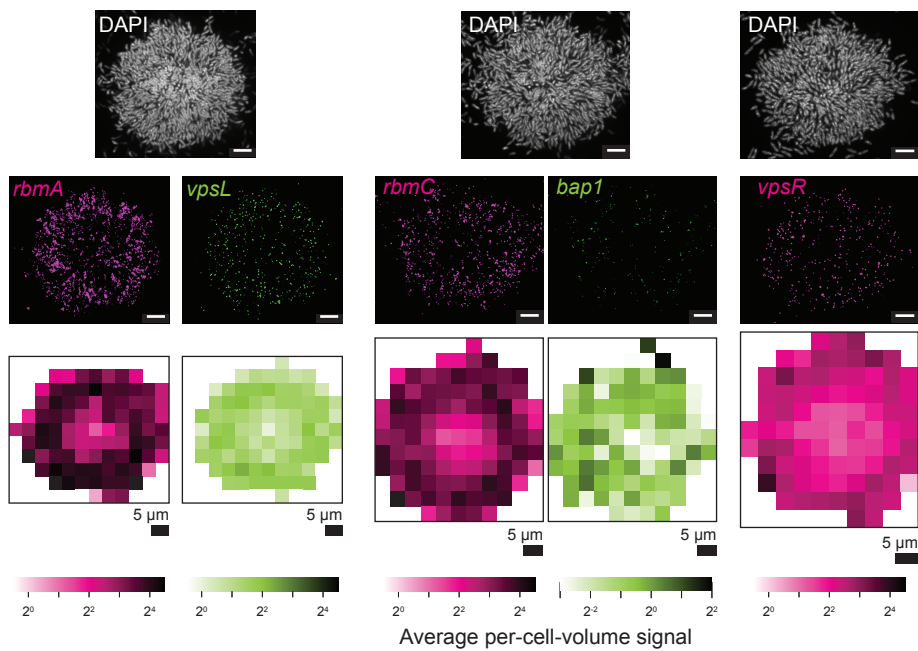

B

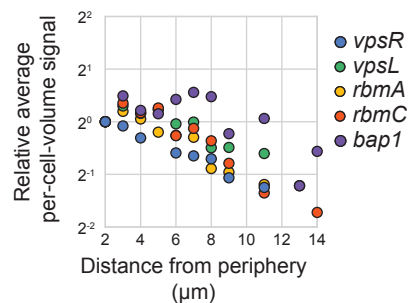

C

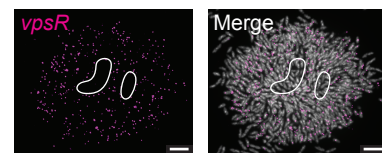

D

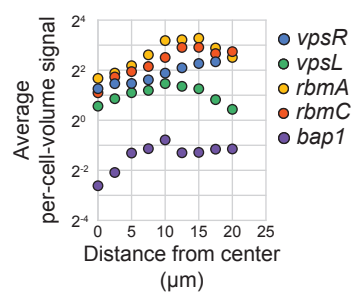

E

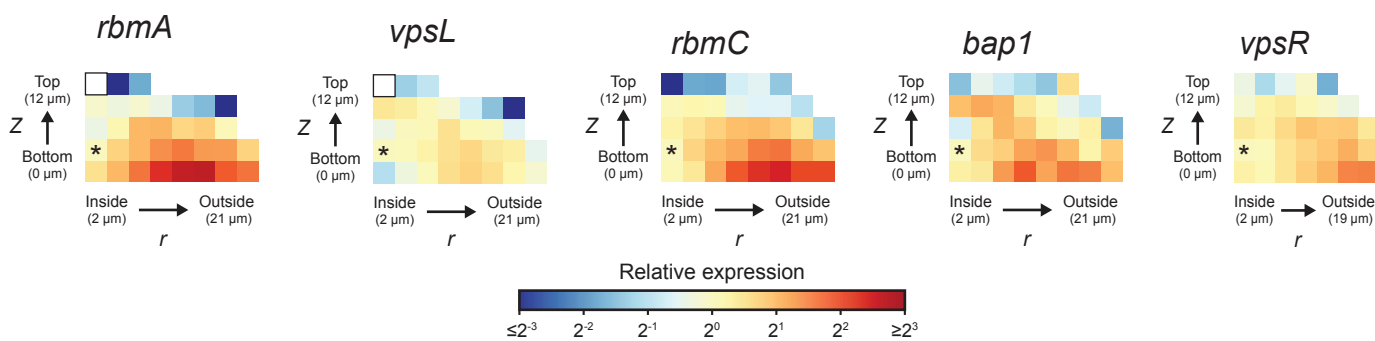

F

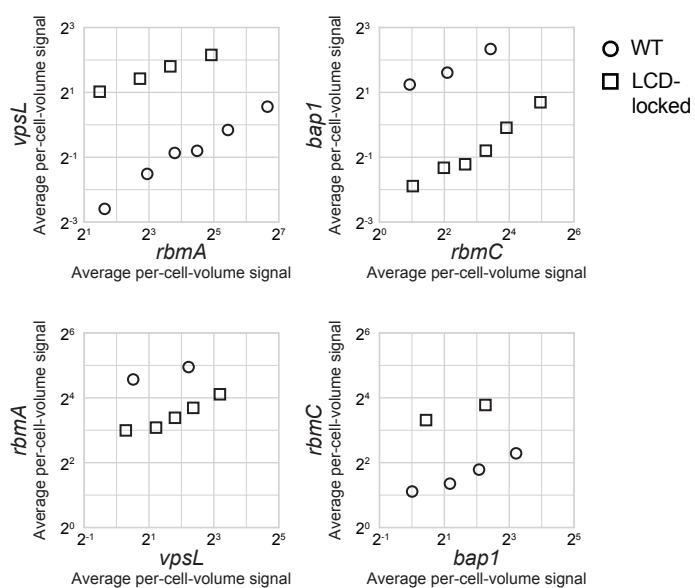

G

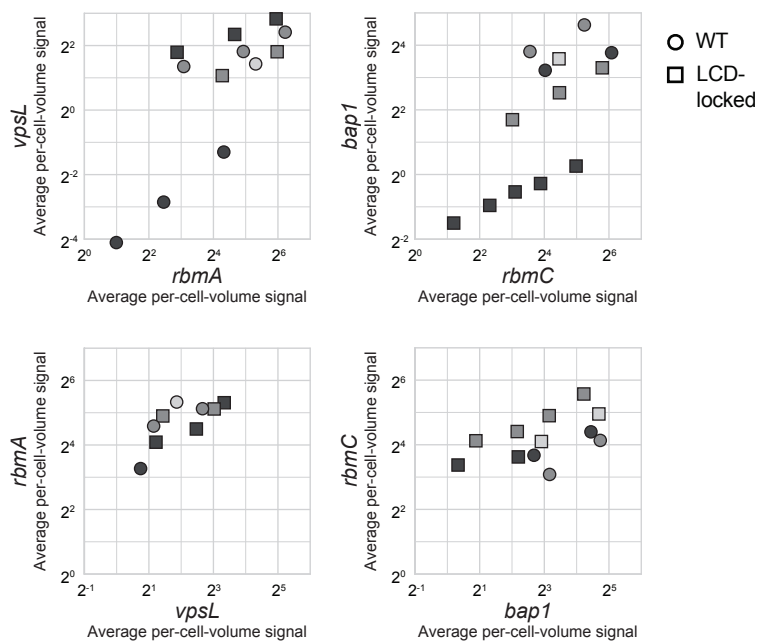

Supplement: S9 Fig — (A) Representative images showing DAPI, and rbmA, rbmC, bap1, and vpsR smFISH fluorescence signals and heatmaps of per-cell-volume fluorescence signal as a function of x, y position as in Fig 3C for n = 5–6 replicate V. cholerae biofilms formed from the LCD-locked strain with the largest biovolumes shown in Fig 6B. Scale bars represent 5 µm. (B) Relative average per-cell-volume fluorescence signal as a function of distance from the periphery of the biofilm as in Fig 3D, for the data shown in (A). (C) Representative images of vpsR smFISH fluorescence signal and merged DAPI and vpsR signal as in (A). Regions containing few cells are outlined. (D) Average per-cell-volume fluorescence signal versus distance to the biofilm center, as in S3A Fig, for the data shown in (E). (E) Heatmaps of rbmA, rbmC, bap1, and vpsR average per-cell-volume fluorescence signal relative to the average per-cell-volume fluorescence signal in the bin denoted with an asterisk are shown as in Fig 2B for n = 5–6 replicate biofilms formed from the LCD-locked strain with the largest biovolume shown in Fig 6B. vpsR appears to be preferentially expressed in cells residing at the periphery. However, the core regions of these particular biofilms contained fewer cells (see S8C Fig) than all other biofilms analyzed in this work and consequently, there was low vpsR smFISH fluorescence signal output from the core. This feature biased the quantitation making it seem as if a spatial pattern exists when, most likely, it does not. (F) Relationship between average per-cell-volume smFISH fluorescence signal between pairs of genes measured in parallel across groups of cells in WT biofilms and biofilms formed by the LCD-locked strain in Figs 6F and S9E, calculated as described in Methods: Correlating gene expression in cell groups. The gene on the x-axis represents the reference gene. (G) As in (F) for additional biofilm sizes. Dark to light shading represents biofilms of decreasing sizes. All data have been correc [file pbio.3003187.s015.pdf]
